# Development of an Automated Chemiluminescent Enzyme Immunoassay for Measuring Thrombopoietin in Human Plasma

**DOI:** 10.3390/diagnostics12020313

**Published:** 2022-01-26

**Authors:** Yukihiro Nishikawa, Shiyo Nishida, Keiko Kuroda, Hirokazu Kashiwagi, Yoshiaki Tomiyama, Masataka Kuwana

**Affiliations:** 1 Medical & Biological Laboratories Co., Ltd., Nagoya 460-0008, Japan; nishikawa.yukihiro@mbl.co.jp (Y.N.); nishida.shiyo@mbl.co.jp (S.N.); kuroda.keiko@mbl.co.jp (K.K.); 2 Department of Hematology and Oncology, Osaka University Graduate School of Medicine, Osaka 565-0871, Japan; kashi@hp-blood.med.osaka-u.ac.jp; 3 Department of Blood Transfusion, Osaka University Hospital, Osaka 565-0871, Japan; yoshi@hp-blood.med.osaka-u.ac.jp; 4 Department of Allergy and Rheumatology, Nippon Medical School Graduate School of Medicine, Tokyo 113-8602, Japan

**Keywords:** thrombopoietin, immunoassay, chemiluminescent, automated, thrombocytopenia, immune thrombocytopenia, aplastic anemia

## Abstract

Plasma thrombopoietin (TPO) measurements help distinguish between different types of thrombocytopenia but are not feasible in routine clinical practice. We developed a fully automated quantitative chemiluminescent enzyme immunoassay (CLEIA) for measuring TPO (TPO-CLEIA), which is a one-step sandwich-type assay. This assay utilizes a mouse monoclonal capture antibody, which has the neutralizing epitope of the interaction between TPO and the TPO receptor, and a newly generated rabbit monoclonal detector antibody. In analytical performance studies, this assay showed good linearity over the measuring range and high sensitivity. The limit of quantification (LoQ) of this assay was 3.4 pg/mL; low TPO concentration values of almost all healthy individuals exceeded the LoQ value. In clinical validation studies, TPO levels obtained from patients with aplastic anemia (AA) significantly increased, whereas those of patients with immune thrombocytopenia (ITP) were normal or slightly increased. The cutoff value for TPO-CLEIA corresponding to the previously reported values was useful for distinguishing between ITP and AA. These results suggest that TPO-CLEIA can quantify human plasma TPO levels with high accuracy and sensitivity and has the potential to facilitate routine clinical measurement of TPO in patients with various types of thrombocytopenia.

## 1. Introduction

Thrombopoietin (TPO) is the primary regulator of platelet production and maturation of megakaryocytes [1]. Previous studies have shown that blood TPO levels are inversely proportional to the number of megakaryocytes in the bone marrow and the circulating platelet count [2,3,4]. These studies have suggested that TPO expression is regulated by a feedback mechanism involving the mass of c-Mpl, which is a TPO receptor present on the surface of platelets and megakaryocytes [5,6,7]. Thus, the measurement of TPO levels may help differentiate between various types of thrombocytopenia.

Immune thrombocytopenia (ITP) is an autoimmune disease characterized by platelet destruction mediated by antiplatelet autoantibodies with or without impaired platelet production [8,9,10]. Recently, platelet desialylation has also been shown to play an important role in platelet destruction and impaired thrombopoiesis in patients with ITP [11]. Despite this progress in understanding pathophysiology, the diagnosis of ITP is still primarily based on differential diagnosis [12]. However, it is sometimes difficult to distinguish ITP from other forms of consumptive thrombocytopenia or hypoplastic thrombocytopenia, including aplastic anemia (AA). Many previous studies have suggested that TPO measurement is helpful in this differential diagnosis since plasma TPO levels are markedly elevated in patients with AA, while they are slightly or not elevated in patients with ITP [13,14,15,16,17,18].

However, TPO measurement is not feasible in routine clinical laboratories because a quantitative assay that combines high sensitivity and high throughput has not been developed yet. The enzyme-linked immunosorbent assay (ELISA) reported by Tanaka et al. is the most sensitive assay and the only one capable of quantifying low TPO levels in healthy individuals [19]. This method utilizes TN1, a monoclonal antibody that has the neutralizing epitope of the interaction between TPO and the TPO receptor as the capture antibody; thus, it is specific to TPO molecules [19,20,21]. However, the assay requires two days to complete and is only used in a handful of clinical laboratories in Japan [22]. Meanwhile, a commonly used commercial kit can complete measurements in half a day; however, according to the manufacturer’s instructions, low TPO levels are undetected in approximately 80% of apparently healthy individuals [15,16,17,18,23]. Furthermore, since the detection antibodies of both reagents are rabbit polyclonal antibodies, the performance quality is difficult to control.

To solve these problems, we developed a fully automated chemiluminescent enzyme immunoassay (CLEIA) based on monoclonal antibodies for measuring TPO (TPO-CLEIA). This assay utilizes a rabbit monoclonal antibody, newly developed using the phage display method, that pairs with TN1. The aim of this study was to evaluate the analytical performance of this assay by measuring plasma TPO levels in healthy individuals and in patients with various types of thrombocytopenia. The secondary aim was to describe the assignment of TPO concentration using the in-house standard material and to compare the measured values with those of a commonly used commercial kit.

## 2. Materials and Methods

### 2.1. Patients

A total of 316 plasma samples from 307 ITP patients and 9 AA patients were obtained from the Keio University Hospital for the secondary use of samples acquired in a previous study [16,17,24]. Primary ITP was defined as thrombocytopenia (platelet counts < 100 × 10^9^/L) for at least 6 months, normal or increased bone marrow megakaryocytes, and no secondary diseases that could account for thrombocytopenia [25]. AA was defined as pancytopenia with hypocellular bone marrow in the absence of an abnormal infiltrate or marrow fibrosis [26]. Patients were enrolled consecutively between January 1997 and December 2014. A total of five high-titer TPO plasma samples in patients with AA were obtained from the Osaka University Hospital. Normal specimens from apparently healthy individuals were obtained from Access Biologicals LLC (Vista, CA, USA). This study was approved by the Ethics Committee at Keio University Hospital (2010-2652) and Osaka University Hospital (16338-3). We obtained informed consent from all patients. This study followed the principles of the Declaration of Helsinki.

### 2.2. Construction of Rabbit Immune Antibody Library

Japanese white rabbits were immunized by a subcutaneous injection of recombinant human TPO protein (100 μg) (rhTPO; rhTPO was specially provided by SRL Inc., Tokyo, Japan), emulsified in complete Freund’s adjuvant. Six subsequent immunizations with 50 μg of rhTPO were given with incomplete Freund’s adjuvant at intervals of 1–2 weeks. Total RNA was extracted from the spleen of the immunized rabbits using ISOGEN (Nippon Gene Co., Ltd., Tokyo, Japan), and cDNA was synthesized with SuperScript^®^ III reverse transcriptase (Thermo Fisher Scientific, Waltham, MA, USA). Using this cDNA, a phage display of single-chain variable fragment (scFv) library was constructed, as described previously [27].

### 2.3. Anti-TPO Antibody Screening Using a Phage Display

The screening of scFv phages that bind to the TPO antigen was performed by the panning method, as described previously [28]. Briefly, the recombinant murine monoclonal anti-TPO antibody (rTN1; MBL, Tokyo, Japan) was immobilized by Dynabeads ProteinG (Thermo Fisher Scientific). In addition, rTN1 was generated by genetic engineering techniques, whereby VH and VL regions of the coding cDNA sequence were synthesized from the TN1 amino acid sequence [21] and fused to murine CH1-3 and CK regions. rhTPO was bound to rTN1 immobilized on the particles, and scFv-displaying phages were exposed to an immunocomplex made of rTN1 and rhTPO. The unbound phages were removed by washing with phosphate-buffered saline (PBS) several times, and the scFv-displaying phages capable of binding to rhTPO via rTN1 were collected. These scFv-displaying phages enriched by TPO-binding activity were rescued from *Escherichia coli* and transformed by the superinfection of bacteria with a helper phage. By repeating these pannings five times, high-affinity scFv-displaying phages were isolated.

### 2.4. Conversion of Anti-TPO Rabbit Monoclonal IgG from Phage Antibodies

Conversion from scFv to rabbit IgG was performed, as described previously [29]. The rabbit IgG type antibody vector was transfected into CHOK1SV GS-KO cells by electroporation. For protein purification, anti-TPO antibody-expressing cells were cultured in CD-CHO medium (Thermo Fisher Scientific). The culture supernatant was collected, and the IgG fraction was obtained using rProtein A Sepharose Fast Flow (Cytiva, Marlborough, MA, USA). Antibody concentration was determined by measuring absorbance at 280 nm. Antibodies specific to rhTPO were screened by ELISA using rhTPO-coated microtiter plates (MaxiSorp™, Thermo). The anti-TPO rabbit monoclonal antibody with the highest affinity for rhTPO (a004-D4) was selected.

### 2.5. Preparation of Alkaline Phosphatase (ALP)-Labeled Anti-TPO Antibody

Alkaline phosphatase (ALP)-labeled anti-TPO antibodies were prepared by conjugating ALP with Fab′ fragments according to the method of Ishikawa et al. [30]. Briefly, a004-D4 F(ab′)2 was prepared by digestion of affinity-purified a004-D4 with pepsin and chromatography of the digest on a HiLoad 26/600 Superdex 200 pg column (Cytiva). It was then converted to Fab’ by reduction with 2-mercaptoethylamine. The resultant a004-D4 Fab′ was conjugated with ALP using the N-hydroxysuccimide ester of N-(carboxycyclohexylmethyl)-maleimide in N, N′-dimethylformamide by the hinge method [30]. The a004-D4 Fab′–ALP conjugate was separated from the mixture by gel filtration on a HiLoad 26/600 Superdex 200 pg column (Cytiva) equilibrated in 1 mM MgCl_2_, 0.1 mM ZnCl_2_, 50 mM Tris-HCl (pH 6.8), and 100 mM NaCl buffer. Fractions containing the a004-D4 Fab′–ALP conjugate were added to bovine serum albumin (BSA) at a final concentration of 1% (*v*/*v*) and stored at 4 °C until use.

### 2.6. Recombinant Human TPO with a C-Terminal 6× His Tag (rhTPO-His) Antigen

Full-length human TPO ORF with a C-terminal 6× His tag, followed by a single stop codon sequence, was synthesized and cloned into pXC17.4 (Lonza Inc., Basel, Switzerland) with HindIII/EcoRI sites, expressed, and purified using the GS Xceed™ Gene Expression System (Lonza). The TPO ORF with a C-terminal 6× His tag in the pXC17.4 vector was transfected into CHOK1SV GS-KO cells by electroporation. For protein purification, TPO-expressing cells were cultured in CD-CHO medium (Thermo Fisher Scientific) for 5 days. The supernatant was collected and loaded onto a HisTrap excel column (Cytiva) equilibrated in 20 mM phosphate (pH 7.4) and 300 mM NaCl buffer. The bound protein was eluted with a phosphate buffer containing 300 mM imidazole. Fractions containing rhTPO-His, as verified by SDS-PAGE, were pooled and loaded onto a 10 mL HiLoad 26/600 Superdex 200 pg column (Cytiva) equilibrated in 2 × PBS. Fractions containing rhTPO-His were added to glycerol at a final concentration of 50% (*v*/*v*) and stored at −80 °C until use.

### 2.7. Measurement of Plasma TPO Concentrations

TPO-CLEIA is a one-step sandwich-type assay. The analyte, TPO, is captured by paramagnetic microparticles coated with rTN1. The analyte–microparticle complex was detected using ALP-labeled a020-D4. Assay calibrators ranged from 0 to 700 pg/mL (0, 20, 70, 200, and 700 pg/mL).

**Figure 1:**
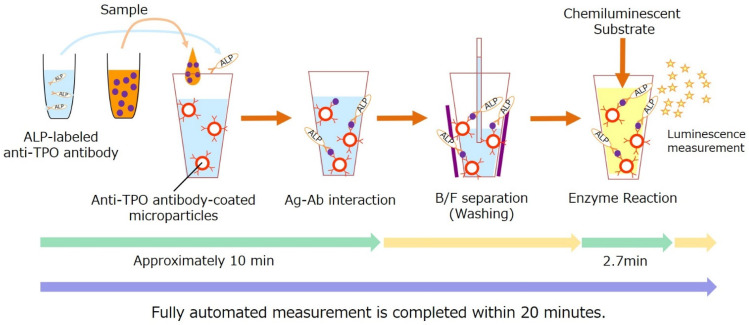
Schematic representation of TPO measurement using a one-step sandwich method. AB, antibody; Ag, antigen; ALP, alkaline phosphatase; TPO, thrombopoietin.

In the first step, 110 μL of anti-TPO antibody-coated microparticles, 40 μL of the sample, and 100 μL of ALP-labeled anti-TPO antibody were mixed and incubated for approximately 10 min. Then, the bound and free fractions were separated, an immunocomplex made of anti-TPO antibody-coated microparticles and TPO and ALP-labeled anti-TPO antibody was incubated with 100 μL of chemiluminescent substrate solution (CDP-Star; Applied BioSystems, Bedford, MA, USA) for 2.7 min, and the luminescence was measured using a luminometer in the STACIA CLEIA system (LSI Medience Corporation, Tokyo, Japan). The assay was fully automated, and measurements were completed within 20 min with a throughput maximum of 270 tests/h. A schematic representation of TPO measurement is shown in Figure 1.

Plasma TPO concentrations measured by TPO-CLEIA were compared to those obtained by TPO-ELISA (Human Thrombopoietin Quantikine ELISA Kit, R&D Systems, Minneapolis, MN, USA), a commercially available kit [23].

### 2.8. Characterization of In-House Standard Material and Concentration Assignment

rhTPO was used as the in-house standard material since there is no international standard for a TPO in vitro diagnostic reagent. The rhTPO concentration was calibrated by BSA standard (Thermo) using the Quick Start™ Bradford Protein Assay (Bio-Rad). The TPO measurement values for TPO-CLEIA were assigned by assay calibrators prepared from rhTPO-His, which is equal to the concentration of rhTPO for the in-house standard material.

### 2.9. Evaluation Methods for TPO-CLEIA

Assay precision was evaluated using intra- and interassay precision methods. For intra-assay precision, three levels of plasma samples were assayed in replicates of five using three lots. Similarly, interassay precision was evaluated using three levels of samples assayed at five separate times using three lots. The intra- and interassay validations used samples with different TPO concentrations.

Dilution linearity studies were conducted with native high-titer plasma specimens and plasma specimens spiked with rhTPO. The specimens were serially diluted in 10% increments with calibrator dilution buffer. Regression analyses of the observed diluted concentrations were compared to the expected values based on the corresponding concentrations of the undiluted specimen. The absence of a high-dose hook effect was confirmed by testing specimens beyond the dynamic range of the assay (700 pg/mL) up to approximately 2300 pg/mL to determine whether signal suppression occurs at analyte levels exceeding the concentration levels of the assay calibration.

Functional and analytical sensitivity were defined as the limit of quantification (LoQ) and the limit of detection (LoD), respectively, which were evaluated using a set of dilution panels prepared by diluting a normal specimen with the calibrator dilution buffer. The panel members were tested in replicates of 8 two times. The LoQ was set as the lowest concentration that showed a total precision of 15% coefficient of variation (%CV). The LoD was set as the minimum value in the dilution panels where the mean+2SD value on the low side did not exceed the mean-2SD value on the high side.

Recovery was evaluated by spiking rhTPO into three EDTA plasma samples. Percent recovery was calculated using the following formula: (% recovery) = ([observed concentration of sample spiked TPO plasma] − [concentration of the unspiked sample])/(TPO concentration added). Note that the calibrator buffer was added to the unspiked sample to maintain the same volume as that of the spiked sample.

The influence of interfering substances was verified using Interference Check A Plus, Interference Check RF Plus (Sysmex, Kobe, Japan), and normal human IgG (FUJIFILM Wako Pure Chemical Corporation, Osaka, Japan). Three levels of plasma samples were mixed with free bilirubin, conjugated bilirubin, hemoglobin, chyle material, rheumatoid factor, or high concentration of normal human IgG (500 mg/dL). The mean TPO value of each test sample was compared with the mean value of the corresponding control sample.

The equivalence of plasma samples from five healthy subjects was evaluated using three different anticoagulants: EDTA, heparin, and citric acid. A total of 50 pg/mL or 200 pg/mL rhTPO was spiked into each specimen and assayed in replicates of three. The mean TPO value of each test sample was compared with the mean value of the corresponding control sample.

### 2.10. Statistical Analysis

All continuous variables are presented as mean ± standard deviation. The differences between mean values were evaluated using Student’s *t*-test, and a *p*-value of <0.001 was considered statistically significant. Statistical analysis was performed using Stat Flex ver.7 (Artech, Osaka, Japan).

## 3. Results

### 3.1. Characterization of Recombinant Human TPO Antigen

**Figure 2:**
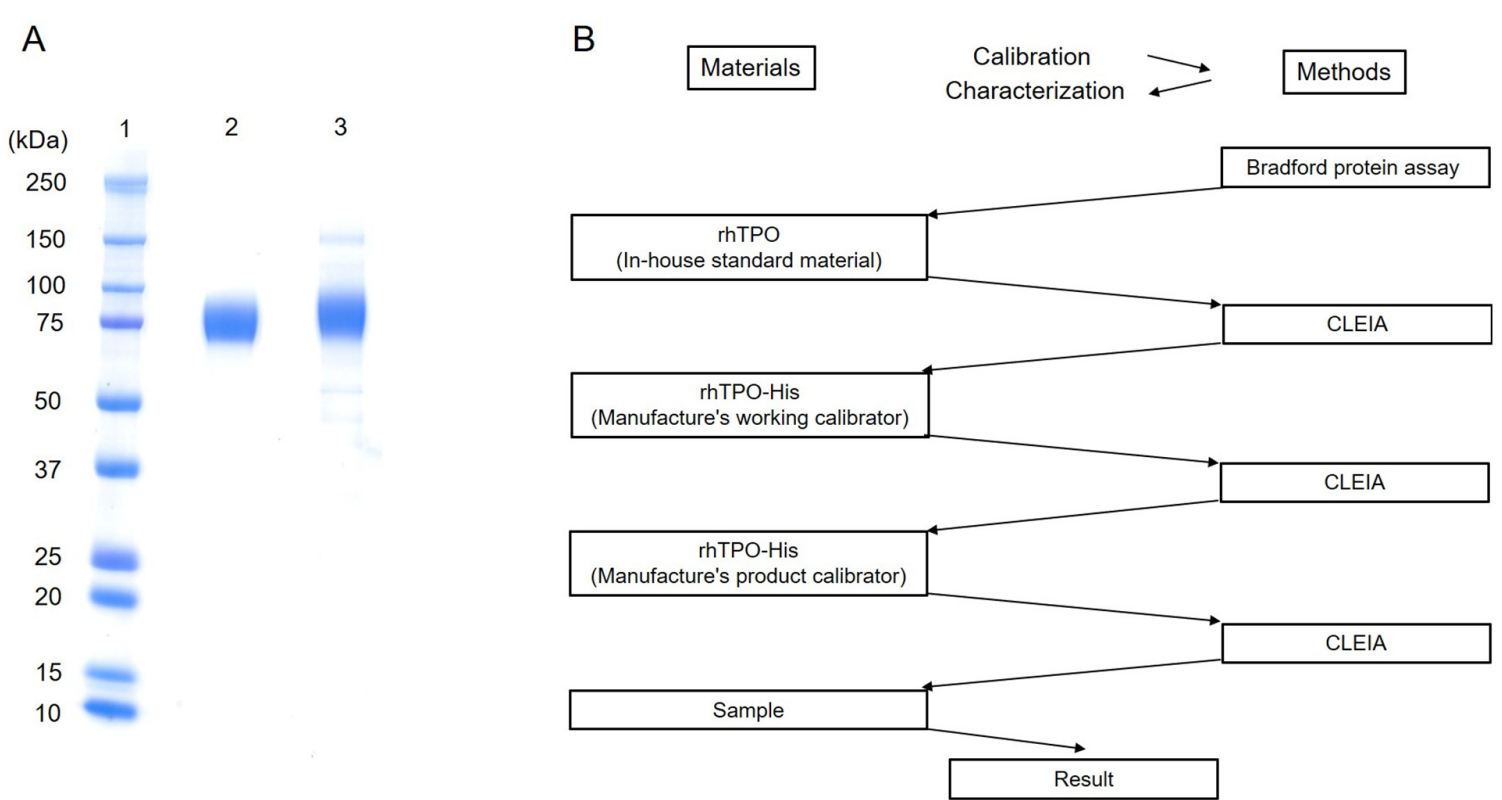
Comparison of purity levels between the isolated rhTPO-His and rhTPO as in-house standard material and traceability system diagram using these materials. (**A**) The samples in lanes 1–3 were subjected to SDS-PAGE. Lane 1, prestained molecular mass markers (Bio-Rad) of indicated size (kDa); 2, rhTPO as in-house standard material; 3, isolated rhTPO-His. Note: 1 µg of total protein was loaded in lanes 2–3. (**B**) Traceability of thrombopoietin (TPO) concentration was ensured by the new CLEIA assay using BSA standard and the Bradford protein assay. BSA, bovine serum albumin; CLEIA, chemiluminescent enzyme immunoassay; rhTPO, recombinant human thrombopoietin.

The purity of the isolated protein was determined by SDS-PAGE and was approximately comparable to that of rhTPO used as in-house standard material (Figure 2A); the same intensity was obtained for each TPO antigen with the same protein concentration. The traceability system diagram of the TPO-CLEIA kit is shown in Figure 2B.

The value of the in-house standard rhTPO (70 pg/mL), calibrated by the BSA standard and measured with TPO-ELISA (R&D Systems), was 300 pg/mL. From this result, it was expected that TPO levels obtained by TPO-CLEIA could be converted to those obtained by TPO-ELISA by multiplying the former value by a coefficient of 4.286 (=300/70); this relationship was confirmed in analyses of some high-titer TPO plasma samples in patients with AA, indicating that TPO-CLEIA accurately measures high TPO concentrations in patients with AA (Table 1). When a dilution series of one of the samples (No. 5) was used, concordant results obtained by TPO-CLEIA and TPO-ELISA were found up to 40-fold dilution, but there was discordance at 80-fold dilution, probably due to low sensitivity of TPO-ELISA.

### 3.2. Analytical Performance of TPO-CLEIA

Assay precision values are shown in Table 2. Intra- and interassay precision values ranged from 0.8% to 4.7% and 1.3% to 5.9%, respectively.

**Figure 3:**
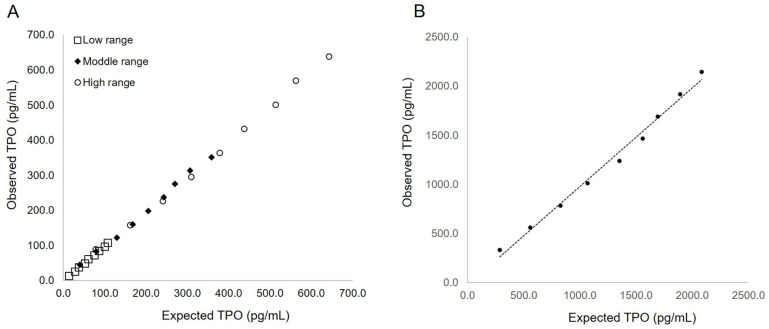
Evaluation of linearity and high-dose hook effect. (**A**) Relationship between expected TPO concentrations calculated from dilution ratio and the observed concentrations in three samples. (**B**) Relationship between expected TPO concentrations and observed high TPO concentrations beyond the dynamic range. TPO, thrombopoietin.

All dilution linearity studies using specimens with concentrations ranging from 13.1 to 684.9 pg/mL showed correlation coefficients ranging from 0.998 to 0.999 (Figure 3A). The recovery rate of the diluted samples ranged from 87.3% to 106.3%. No high-dose hook effect was observed in the testing conditions up to approximately 2300 pg/mL (Figure 3B). Under these conditions, the dilution linearity showed a correlation coefficient of 0.996 and recovery rates in the range of 86.0% to 109.0%.

**Figure 4:**
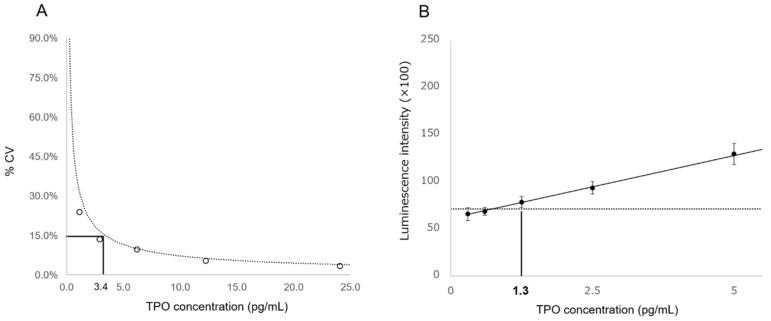
Results of plasma thrombopoietin functional sensitivity and analytical sensitivity studies. (**A**) The concentration, 3.4 pg/mL, was set as the lowest concentration that showed a total precision of 15% CV. (**B**) The concentration, 1.3 pg/mL, was set as the minimum value in the dilution panels where the mean + 2SD value on the low side did not exceed the mean-2SD value on the high side. CV, coefficient of variation; TPO, thrombopoietin.

From the LoQ results, the functional sensitivity at 15% CV was 3.4 pg/mL (Figure 4A); based on these results, the dynamic range for this reagent was defined as 3.4–700 pg/mL. From the LoD results, the analytical sensitivity was 1.3 pg/mL (Figure 4B).

The recovery rates obtained after spiking rhTPO into three plasma samples are shown in Table 3. The percentage recovery ranged from 86.8% to 106.0%.

The influence of interfering substances is shown in Table 4. The mean percentage differences between the test samples and control samples ranged from −8.0% to 4.0%.

The evaluation of plasma sample type equivalence using EDTA, heparin, and citrate is shown in Table 5. The average percentage difference ranged from −8.0% to 11.6%.

### 3.3. Clinical Validation in the Study Participants

**Figure 5:**
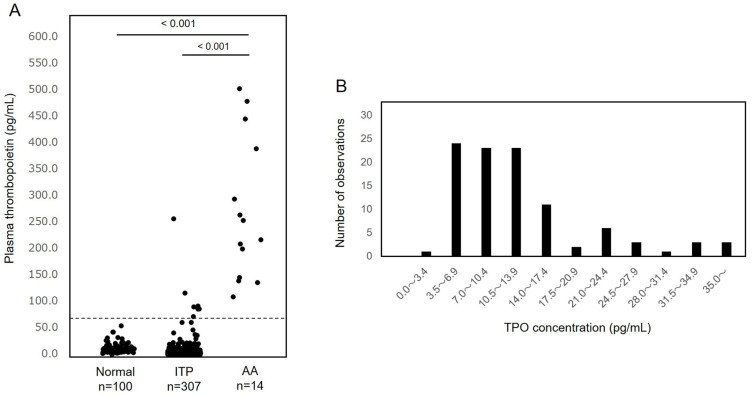
Clinical validation of TPO-CLEIA in study participants. (**A**) Plasma thrombopoietin (TPO) levels in healthy controls, patients with immune thrombocytopenia (ITP), aplastic anemia (AA). (**B**) Distribution of TPO concentrations in healthy controls.

We quantified the TPO levels in plasma samples from 100 healthy controls, 307 ITP patients, and 14 AA patients using TPO-CLEIA (Figure 5A). The TPO levels obtained from 100 healthy controls were 12.8 ± 9.0 pg/mL (mean ± SD). Patients with AA showed markedly increased (271.0 ± 133.5 pg/mL) plasma TPO levels, and those with ITP showed normal or slightly increased (9.6 ± 20.3 pg/mL) plasma TPO levels. The upper limit of the reference range of patients with ITP was 70.4 pg/mL (mean + 3SD). This measurement value was 300 pg/mL when converted to TPO-ELISA values, which corresponds to the cutoff value useful for distinguishing between ITP and AA [16].

The distributions of TPO concentrations in healthy controls measured by TPO-CLEIA are shown in Figure 5B. As shown in Figure 5B, in almost all of them, the measurement values were above 3.4 pg/mL (LoQ), except for one, which was remarkably low.

## 4. Discussion

In this study, we report the development of a high-throughput, highly specific TPO-CLEIA via the STACIA system, which is used in clinical laboratories. To achieve maximum sensitivity and throughput, we established the monoclonal antibody a020-D4 as a detector, which was found to be the optimal combination for the monoclonal antibody rTN1 as a capture antibody. As a result, the new system achieved full automation, and the measurement was completed within 20 min with a throughput maximum of 270 tests/h.

The standardization of measurements contributes significantly to improving healthcare by producing universally applicable results of clinical studies conducted at different locations or times [31,32]. This enables the effective application of evidence-based medicine and the use of established guidelines for diagnostic and therapeutic interventions. To achieve standardization, an approach is needed to ensure that measurement values are transferred from the highest hierarchical level to the methods routinely used in clinical laboratories [31,33]. One of the essential elements of this approach is the establishment of international reference standard materials [31], but TPO measurements do not yet have one for in vitro diagnostic use. Nevertheless, many studies using the TPO-ELISA kit have been reported, but information on the purity of the standard materials included in the TPO-ELISA kit is not provided. Therefore, we used highly pure rhTPO as in-house standard material and developed rhTPO-His of approximately the same purity as that of rhTPO (see Figure 2A). The TPO measurement values for TPO-CLEIA were assigned by assay calibrators prepared from rhTPO-His, which is equal to the concentration of rhTPO for the in-house standard material. In this way, the obtained TPO value can be traceable to the reference materials. We show that plasma TPO values can be converted to TPO-ELISA-based values by multiplying the TPO-CLEIA-based values by a coefficient of 4.286. These results suggest that the purity of the standard materials included in the TPO-ELISA kit is very low. Nevertheless, the findings obtained by the TPO-ELISA kit in previous studies can be compared with those obtained by TPO-CLEIA.

Moreover, we evaluated the analytical performance of TPO-CLEIA in several ways. The assay demonstrated good precision and good linearity over the measuring range, with no hook effect up to approximately 2300 pg/mL. According to previous studies, the TPO concentration of patients with AA was distributed within 3000 pg/mL, as measured by TPO-ELISA, suggesting that TPO-CLEIA can measure high-titer TPO plasma samples within the dynamic range (approximately 700 pg/mL) [34]. The assay demonstrated that there was no interference with the measurement value by endogenous substances, high concentrations of IgG, or different anticoagulants. However, for consistency with our previous clinical studies [17,18], we recommend that plasma samples used for the TPO-CLEIA assay be treated with EDTA as an anticoagulant. The functional sensitivity was defined by an LoQ of 3.4 pg/mL, which is equal to or higher than the sensitivity of the most sensitive ELISA reported by Tanaka et al. [19]. Using TPO-CLEIA, we showed that the TPO levels in plasma samples from healthy controls and patients with thrombocytopenia could be quantified accurately and sensitively. Notably, the TPO levels in 99% of healthy controls were confirmed to be above the functional sensitivity threshold; we propose that TPO-CLEIA is capable of quantifying low TPO levels in healthy individuals. In contrast, TPO-ELISA has been shown to be undetectable in about 80% of healthy individuals, according to the manufacturer’s instructions. Therefore, we tried to calculate LoQ and LoD in TPO-ELISA using the same method as we used in TPO-CLEIA and found an LoQ of 40.7 pg/mL and LoD of 21.4 pg/mL, corresponding to 9.5 pg/mL and 5.0 in TPO-CLEIA, respectively. According to the manufacturer’s instructions, the sensitivity of TPO-ELISA is 18.4 pg/mL. The method of calculating this “sensitivity” is similar to that of calculating LoD in this study, and the difference between 21.4 pg/mL and 18.4 pg/mL is negligible. However, the LoQ and LoD of TPO-ELISA shown here are our own calculated values and do not guarantee the performance of this kit.

Table 6 shows that TPO-CLEIA is superior to TPO-ELISA in terms of sensitivity, dynamic range, measurement time, and user-friendliness. In general, the CLEIA method is known to have better analytical sensitivity than ELISA and has many advantages, such as low interference emission (that is, high specificity), rapid acquisition of analytical signals, and random access. Fully automated random-access processing allows for quick turnaround times and increased flexibility of routines in clinical laboratories with varying requirements [35]. Rapid access to test results is vital in clinical practice, and clinical laboratories are increasingly shifting to fully automated random-access systems focused on bead-based chemiluminescent technology [35]. The new fully automated TPO-CLEIA that we have developed can meet these clinical laboratory requirements and is expected to be used in the future for diagnosis and the determination of therapeutic effects.

Interestingly, previous studies have shown that serum thrombopoietin levels in very low concentrations decrease slightly as the disease progresses from mild fibrosis to cirrhosis of the liver [36]. Since TPO-CLEIA can accurately measure low TPO levels, it may be possible to closely monitor small changes in TPO concentration during the progression of liver cirrhosis. Thus, it is expected that the extremely low concentration of TPO measurement by the highly sensitive TPO-CLEIA will gain new clinical significance not only in thrombocytopenia but also in new disease areas.

In clinical validation studies, TPO-CLEIA assessments can distinguish between ITP and AA at a cutoff value of 70 pg/mL, corresponding to 300 pg/mL in TPO-ELISA measurements, as reported in previous studies [16]. TPO measurements were above the cutoff value for seven ITP cases, but these cases also had higher TPO concentrations for TPO-ELISA measurements (data not shown). Further clinical research is required to confirm that this TPO test can be used for the diagnosis of ITP and AA, and eventually thrombocytopenia, in general clinical practice.

In conclusion, TPO-CLEIA demonstrated the convenience, high throughput, and good analytical performance necessary for TPO measurements to aid in the differential diagnosis of ITP and AA in patients with thrombocytopenia. In addition, this assay provides a convenient automated method for measuring plasma TPO levels in hospitals and clinical laboratories. Additional clinical studies in Japan using TPO-CLEIA via the STACIA CLEIA system are currently underway to enable its use to manage patients with various types of thrombocytopenia.

## Figures and Tables

**Table 1:** Correspondence of measured values in TPO-CLEIA and TPO-ELISA.

High-Titer TPO Plasma Samples from AA Patients	TPO-CLEIA Value (pg/mL)	Multiplied by the Coefficient *	TPO-ELISA Value (pg/mL)	% Difference
No. 1	294.8	1263.7	1242.9	2.0
No. 2	200.1	857.7	896.1	−0.4
No. 3	446.3	1912.8	2083.6	−0.8
No. 4	479.6	2055.4	2155.3	−0.5
No. 5	503.6	2158.5	2142.6	1.0
10-fold dilution of No. 5	52.7	225.9	240.1	−5.9
20-fold dilution of No. 5	26.6	114.0	120.0	−5.0
40-fold dilution of No. 5	12.8	54.6	58.6	−6.7
80-fold dilution of No. 5	6.2	26.4	18.5	42.7

* Coefficient: 4.286. AA, aplastic anemia; CLEIA, chemiluminescent enzyme immunoassay; ELISA, enzyme-linked immunosorbent assay; TPO, thrombopoietin.

**Table 2:** Intra- and interassay validations.

TPO Level of Samples	Precision	Intra-Assay Precision			Interassay Precision		
	Reagent Lot	Lot.1	Lot.2	Lot.3	Lot.1	Lot.2	Lot.3
Low level	Mean (pg/mL)	52.2	50.7	52.2	46.8	45.9	47.1
	SD (pg/mL)	1.3	1.3	0.9	1.3	1.5	1.1
	CV	2.5%	2.5%	1.8%	2.9%	3.2%	2.3%
Middle level	Mean (pg/mL)	127.3	121.2	126.1	108.2	106.7	106.8
	SD (pg/mL)	3.1	5.7	1.0	4.6	2.0	1.4
	CV	2.4%	4.7%	0.8%	4.3%	1.9%	1.3%
High level	Mean (pg/mL)	326.0	314.3	331.5	309.2	288.6	286.7
	SD (pg/mL)	6.3	12.3	8.2	5.6	6.7	16.9
	CV	1.9%	3.9%	2.5%	1.8%	2.3%	5.9%

SD: standard deviation; CV: coefficient of variation; TPO: thrombopoietin.

**Table 3:** Recovery of TPO spiked into plasma samples.

TPO Level of Samples	Unspiked Samples (pg/mL)	Spiked TPO (pg/mL)	Observed (pg/mL)	Recovery
Low level	41.8	49.3	87.9	93.5%
		137	181	101.6%
		333.9	349.6	92.2%
Middle level	148.1	49.3	194.9	95.0%
		137	282.6	98.2%
		333.9	437.9	86.8%
High level	398.4	49.3	450.6	106.0%
		137	539.3	102.9%
		333.9	719.4	96.2%

TPO, thrombopoietin.

**Table 4:** Evaluation of interfering substances.

Interfering Substances	Concentration	% Difference from Control		
		TPO Level of Samples		
		Low	Middle	High
Hemoglobin	500 mg/dL	2.3	0.7	4.0
Free bilirubin	20 mg/dL	−2.1	2.1	−8.0
Conjugated bilirubin	20 mg/dL	−2.3	−0.4	−2.6
Chyle material	1360 FTU	−2.7	0.7	−0.9
Rheumatoid factor	500 IU/mL	1.2	0.4	−2.4
High concentration of IgG	500 mg/dL	−6.5	0.2	−6.9

IgG, immunoglobulin gamma; TPO, thrombopoietin.

**Table 5:** Evaluation of plasma sample type equivalence using different anticoagulants.

Sample Type	% Difference from Control		Average Difference, %
	Spiked TPO (pg/mL)		
	50 pg/mL, %	200 pg/mL, %	
EDTA plasma-1	2.3	−6.0	−0.9
EDTA plasma-2	11.6	−3.3	
EDTA plasma-3	−1.6	−5.9	
EDTA plasma-4	4.4	−1.1	
EDTA plasma-5	−1.4	−7.7	
Heparin plasma-1	10.5	−1.1	2.2
Heparin plasma-2	11.7	1.6	
Heparin plasma-3	−6.8	−6.1	
Heparin plasma-4	8.7	0.7	
Heparin plasma-5	6.1	−3.1	
Citrate plasma-1	1.1	−8.0	−0.7
Citrate plasma-2	7.9	−4.2	
Citrate plasma-3	1.8	−5.9	
Citrate plasma-4	10.5	−0.9	
Citrate plasma-5	−2.1	−6.7	

TPO, thrombopoietin.

**Table 6:** Comparisons of features and performance between assays used for quantitative TPO measurements.

Principle of Measurement	CLEIA	ELISA
Trade name	Under consideration	Human Thrombopoietin Quantikine ELISA Kit
Manufacturer	Medical & Biological Laboratories Co., Ltd.	R & D Systems
Capture antibody	Monoclonal	Monoclonal
Detection antibody	Monoclonal	Polyclonal
Operation	Fully automated	Hand method
Measurement time	20 min	4.5 h
Sample volume	40 μL	200 μL
LoQ	14.6 * (3.4) pg/mL	40.7 pg/mL
LoD	5.6 * (1.3) pg/mL	21.4 pg/mL
Upper limit of measurement	3000 * (700) pg/mL	2000 pg/mL

* This value is converted to the ELISA value by multiplying the CLEIA value by a coefficient of 4.286. CLEIA, chemiluminescent enzyme immunoassay; ELISA, enzyme-linked immunosorbent assay; LOD, limit of detection; LOQ, quantification limit.

## Data Availability

Raw data are available from the corresponding author upon reasonable request.
